# *Pleurothallis pembertonii* (Orchidaceae, Pleurothallidinae), a new species from northwestern Ecuador in the Tropical Andes Biodiversity Hotspot

**DOI:** 10.3897/phytokeys.276.190930

**Published:** 2026-06-26

**Authors:** Marco F. Monteros, Kevin Holcomb, Luis E. Baquero, Mark Wilson

**Affiliations:** 1 Fundación EcoMinga, Valladolid N24-414 y Luis Cordero, Quito, Pichincha, Ecuador Grupo de Investigación en Biodiversidad, Medio Ambiente y Salud (BIOMAS), Carrera de Ingeniería en Agroindustria, Facultad de Ingeniería y Ciencias Aplicadas, Universidad de Las Américas, UDLA Quito Ecuador; 2 Reserva The Youth Land Trust, Washington, D.C., USA Unidad de Investigación, Instituto Nacional de Biodiversidad Quito Ecuador; 3 Grupo Científico Calaway Dodson: Investigación y Conservación de Orquídeas del Ecuador, Quito, 170510, Pichincha, Ecuador Department of Organismal Biology and Ecology, Colorado College Colorado Springs United States of America; 4 Unidad de Investigación, Instituto Nacional de Biodiversidad, Quito, Ecuador Grupo Científico Calaway Dodson: Investigación y Conservación de Orquídeas del Ecuador Quito Ecuador; 5 51445 Monroe Dr. E27, Atlanta GA 30324, USA Fundación EcoMinga Quito Ecuador; 6 Grupo de Investigación en Biodiversidad, Medio Ambiente y Salud (BIOMAS), Carrera de Ingeniería en Agroindustria, Facultad de Ingeniería y Ciencias Aplicadas, Universidad de Las Américas, UDLA, Vía a Nayón, Quito 170124, Ecuador Reserva The Youth Land Trust Washington United States of America; 7 Department of Organismal Biology and Ecology, Colorado College, Colorado Springs, CO 80903, USA Unaffiliated Atlanta United States of America

**Keywords:** *
Amphigyae
*, Andean cloud forest, endemism, mining threat

## Abstract

A new species of *Pleurothallis* (Orchidaceae, Pleurothallidinae) from the lower montane cloud forests of northwestern Ecuador is described and illustrated. *Pleurothallis pembertonii* M.F.Monteros & Mark Wilson, **sp. nov**., belongs to *Pleurothallis subgen. Pleurothallis sect. Pleurothallis*, subsect. *Acroniae*, ser. *Amphigyae*, and is most similar in appearance to *P. forceps-cancri*, from which it can be readily distinguished by its vinaceous, oblong-subsigmoid petals that are long-attenuate and by a sagittate-trilobed lip bearing a well-developed reniform glenion and two small divergent basal keels. The new species is currently known only from a single locality within the Dracula Reserve, in the western Andean foothills of Carchi Province, a region characterized by high levels of microendemism and increasing pressure from mining activities.

## Abstract (es)

Se describe e ilustra una nueva especie de *Pleurothallis* (Orchidaceae: Pleurothallidinae) de los bosques nubosos montanos bajos del noroeste de Ecuador. *Pleurothallis pembertonii* M.F.Monteros & Mark Wilson, **sp. nov**. pertenece al subgen. *Pleurothallis*, sect. *Pleurothallis*, subsect. *Acroniae*, ser. *Amphigyae* y es morfológicamente similar a *P. forceps-cancri*, de la cual se distingue fácilmente por sus pétalos vinosos, oblongo-subsigmoides y alargados-atenuados, y por un labelo sagitado-trilobulado que presenta un glenion reniforme bien desarrollado y dos pequeñas quillas basales divergentes. La nueva especie se conoce actualmente solo de una localidad dentro de la reserva Drácula, en las estribaciones andinas occidentales de la provincia de Carchi, una región caracterizada por altos niveles de microendemismo y por la creciente presión derivada de actividades mineras.

## Introduction

*Pleurothallis* R.Br. is among the largest orchid genera in the Neotropics, encompassing 602 accepted species distributed from Mexico to South America and reaching its highest diversity in the northern Andes of Ecuador and Colombia (Pridgeon 2005; Zambrano et al. 2017; Vélez-Abarca et al. 2022; POWO 2026). Despite this extraordinary richness, species delimitation remains challenging in several lineages due to extensive morphological convergence and overlap. One such example is *Pleurothallis subsect. Acroniae ser. Amphigyae*, a small assemblage currently comprising nine accepted species (Luer 1986, 1998; Moreno et al. 2022; POWO 2026). The strong resemblance among its members has historically led to taxonomic confusion and recurrent misidentifications (Luer 1986, 1998). Although some authors have proposed infrageneric rearrangements, including the recognition of *Acronia* as a distinct genus, molecular phylogenetic evidence consistently supports the inclusion of these taxa within a broadly circumscribed *Pleurothallissensu lato* (Pridgeon 2005; Wilson et al. 2011; Karremans 2016).

In recent decades, taxonomic challenges within *Pleurothallis* and other Pleurothallidinae have been exacerbated by the international orchid trade, which has facilitated the circulation of undescribed taxa and specimens identified under misapplied names of morphologically similar species. In multiple instances, new species have been recognized only after flowering in private collections, frequently originating from plants acquired through commercial nurseries and lacking precise or verifiable locality data (Baquero and Mogrovejo 2021; Holcomb 2022, 2023a, 2023b, 2023c; Parra-Sánchez et al. 2024; Baquero et al. 2025).

**Figure 1.**
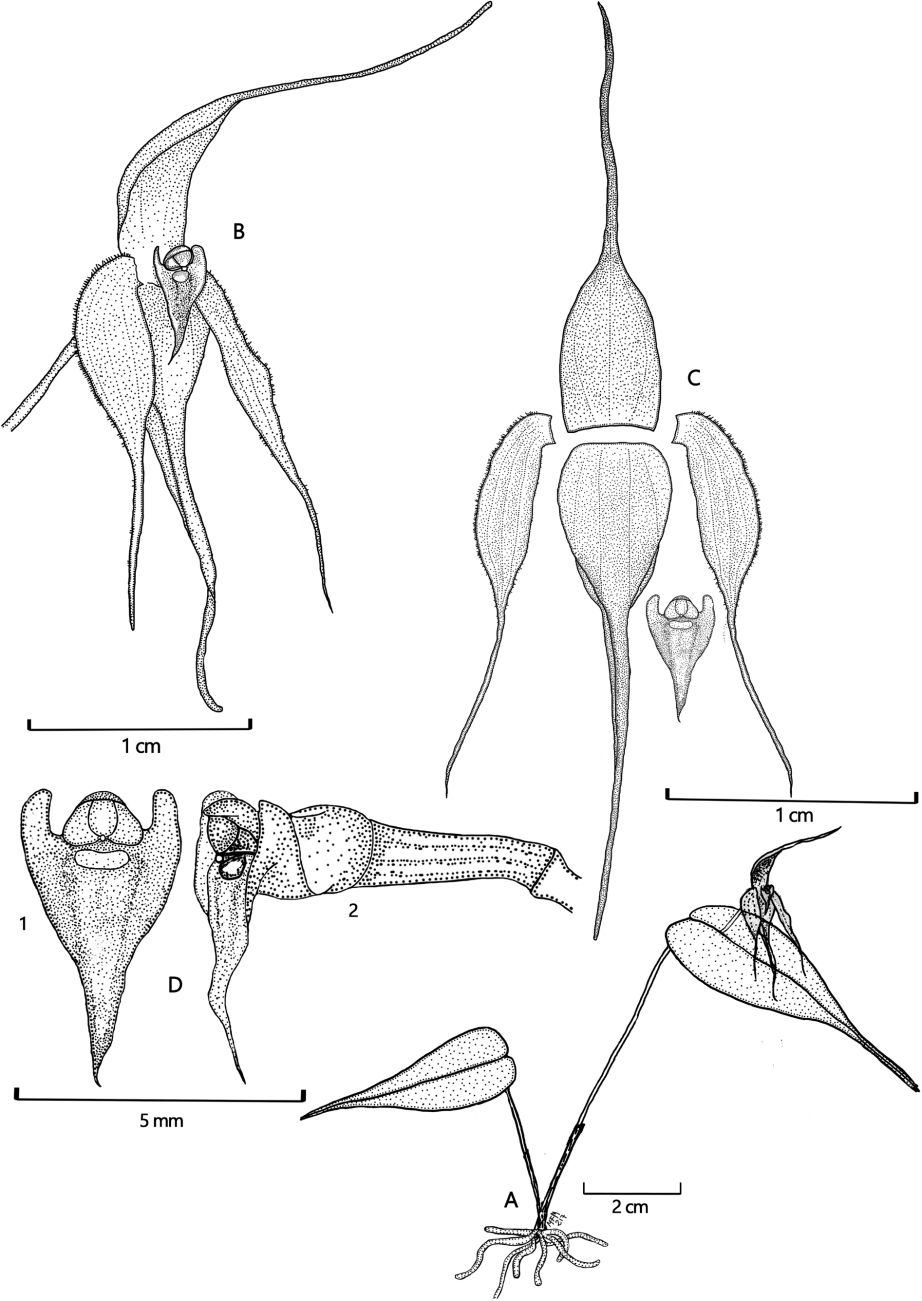
Illustration of *Pleurothallis pembertonii* M.F.Monteros & Mark Wilson. **A**. Habit. **B**. Flower. **C**. Dissected perianth. **D1**. Lip, frontal view. **D2**. Lip, column, and ovary, lateral view. Drawn by Marco F. Monteros from the plant that served as the holotype (MFM 217, QCNE).

**Figure 2.**
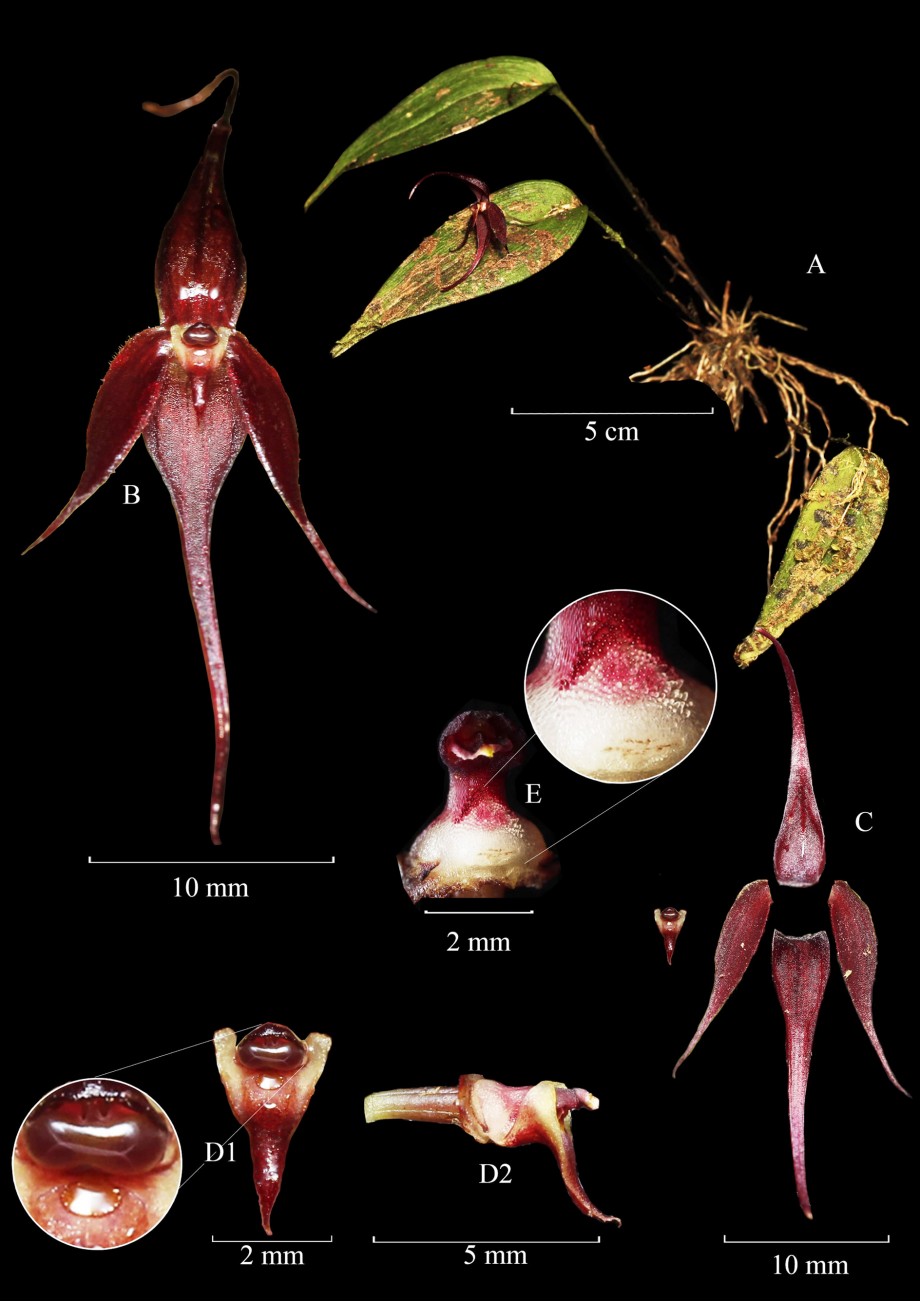
*Pleurothallis pembertonii* M.F.Monteros & Mark Wilson. **A**. Habit. **B**. Flower. **C**. Dissected perianth. **D1**. Lip and stigma, frontal view. **D2**. Column and lip, lateral view, and longitudinal section of the column. **E**. Column, ventral and lateral views. LCDP by Marco F. Monteros based on the paratype (MFM 350).

**Figure 3.**
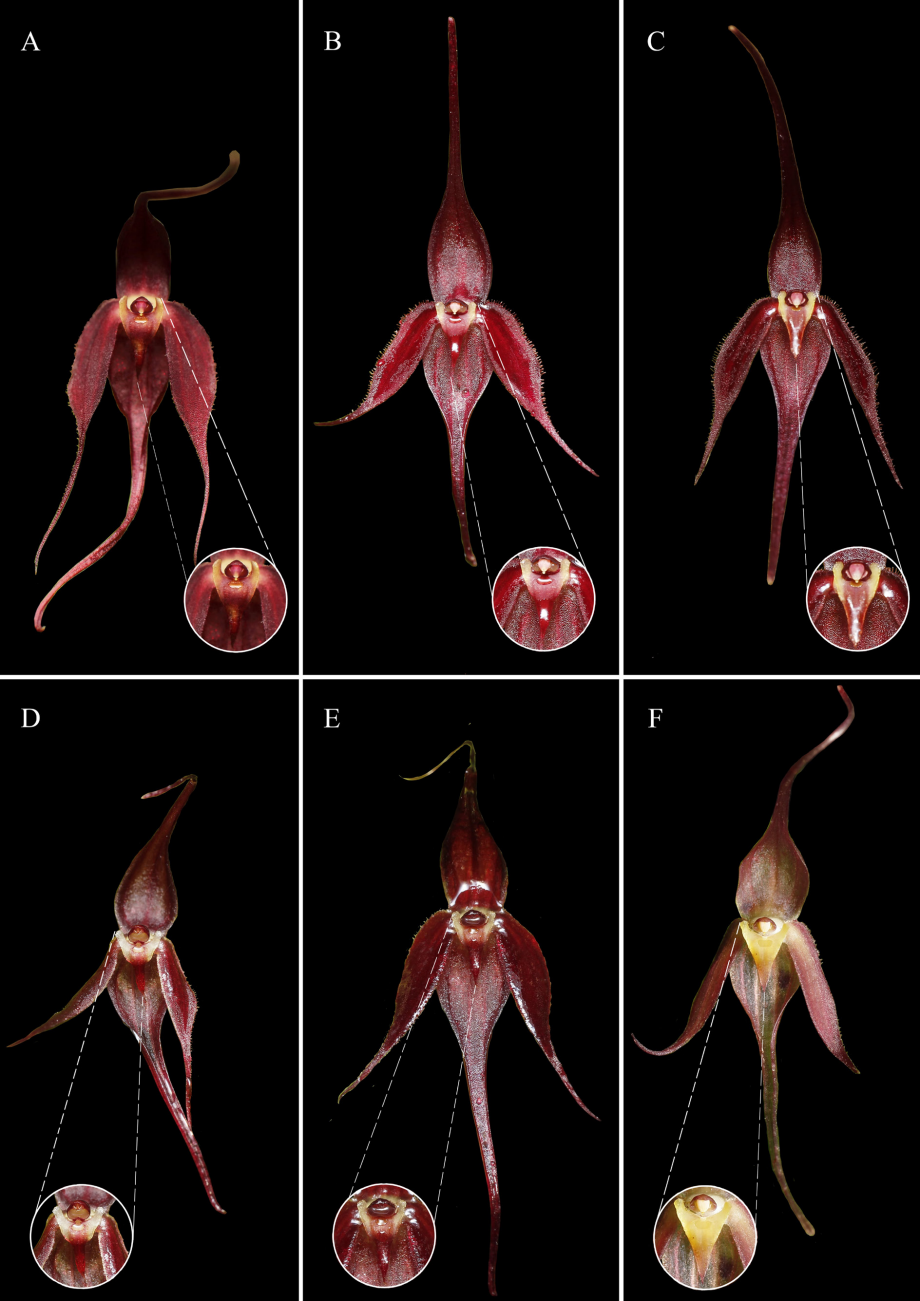
Floral variation in *Pleurothallis pembertonii* M.F.Monteros & Mark Wilson. **A–F**. Flowers from different individuals. Insets show details of the sagittate-trilobed lip with reniform glenion and two small divergent basal keels, which remain constant across all examined individuals.

**Figure 4.**
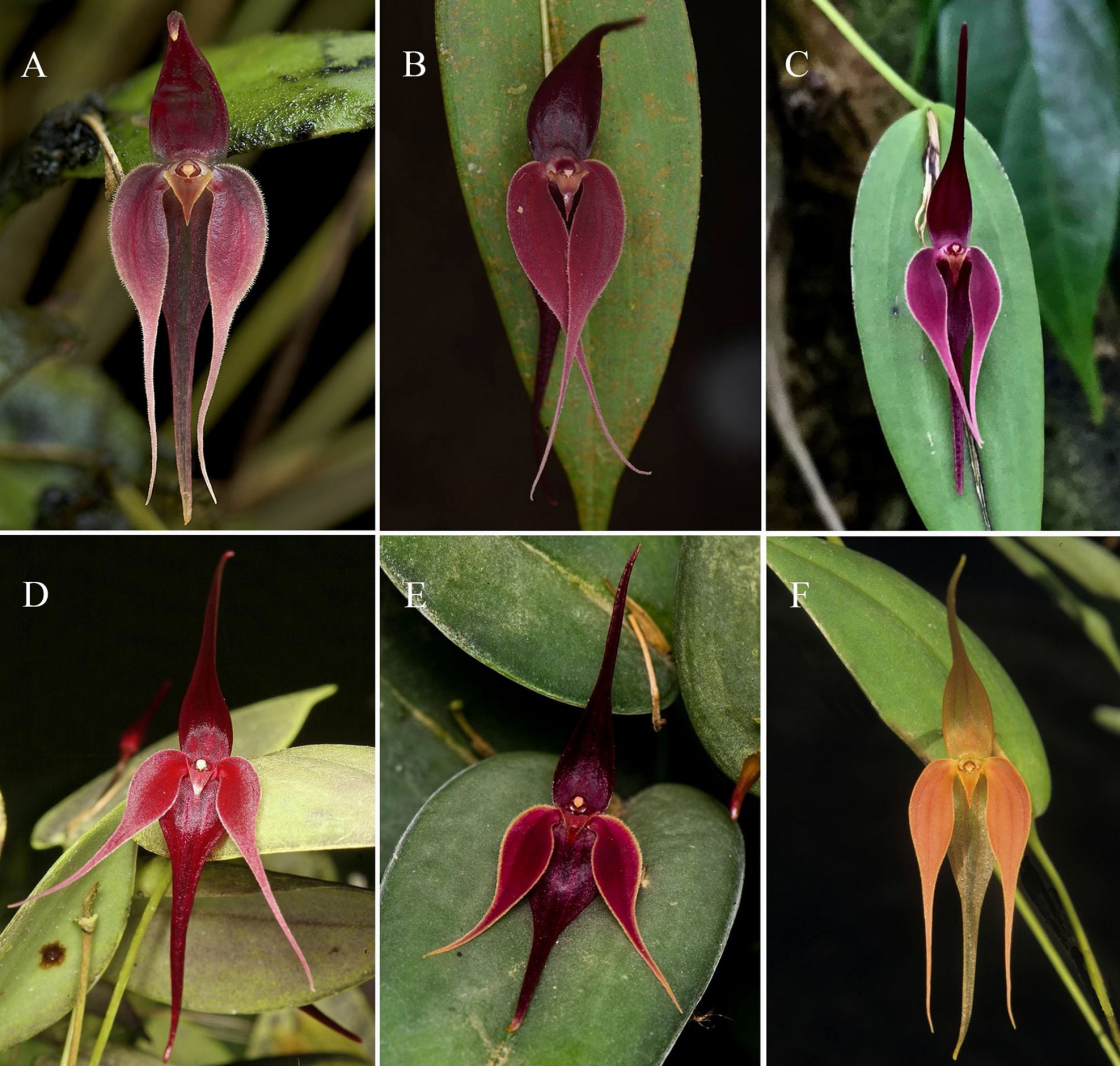
Floral variation in *Pleurothallis forceps-cancri* Luer & R.Escobar. Photographs by **A**. Dale Borders; **B**. Esteban Domínguez; **C**. Kevin Holcomb; **D**. Lourens Grobler; **E**. Luis Eduardo Mejía; **F**. Steve Manning.

The consequences of this process extend well beyond nomenclature and have direct implications for conservation. Species described without reliable provenance remain effectively invisible to spatial analyses, conservation planning, and threat assessments, while their continued circulation under incorrect names may promote unsustainable extraction from unknown or highly restricted wild populations (Parra-Sánchez et al. 2024; Baquero et al. 2025). The recurrent confusion between undescribed taxa and commercially named species highlights the urgent need for integrative, field-based taxonomic approaches. In this study, *Pleurothallis pembertonii* is described and illustrated (Figs 1, 2, 3) as a new species that has been consistently misidentified as *Pleurothallis forceps-cancri* Luer & R.Escobar (Fig. 4) or *P. amphigya* Luer & R.Escobar in horticultural collections, online databases, and photographic records (Kay 2013, 2014; OrchidsSpecies.com 2025; Storczyki Zamość 2025). Data on its morphology, ecology, distribution, and conservation status are also provided.

## Materials and methods

The material of the new species was photographed *in situ* during a scientific expedition to the Dracula Reserve in 2022 using a Canon EOS T6 camera equipped with a Canon EF-S 35 mm f/2.8 macro lens. Voucher specimens were collected and deposited at the Ecuadorian National Herbarium (**QCNE**) under Research Permits No. MAAE-ARSFC-2021-1102 and MAATE-DBI-CM-2021-0187. Additional photographic documentation of fertile plants and fresh flowers was obtained under controlled conditions to support detailed morphological analyses.

Floral material was preserved in 70% ethanol with 1% glycerol and examined in detail under a stereomicroscope. The morphology of the new species was compared with the original descriptions, illustrations, and available type material of morphologically similar species, particularly those belonging to *Pleurothallis subsect. Acroniae ser. Amphigyae* (Luer and Escobar 1981; Luer 1986, 1998; Moreno et al. 2022). A Lankester Composite Digital Plate (**LCDP**) was prepared from high-resolution photographs of the habit, flower, dissected perianth, column, and other diagnostic structures. The plate was assembled in Adobe Photoshop® 2019 following the standardized LCDP protocol of Karremans et al. (2024), including the use of a uniform black background, calibrated scale bars, and the systematic arrangement of morphological features.

Species descriptions follow the botanical terminology of Beentje (2012), as well as the inflorescence terminology proposed by Rojas-Alvarado and Karremans (2024). The conservation status of the new species was assessed according to the IUCN Red List Categories and Criteria (IUCN 2024). The area of occupancy (AOO) was estimated using GeoCAT (Bachman et al. 2011) with the IUCN-recommended 2 × 2 km grid cells. Extent of occurrence (**EOO**) could not be meaningfully estimated because all known records originate from a single locality.

To reduce collection pressure on this narrowly distributed species, precise geographic coordinates are omitted from the published record. Detailed locality information is retained in the herbarium specimen data. Distribution and threat maps were generated using ArcMap v.10. Verified occurrence records were compiled and integrated with thematic layers of vegetation cover, protected areas, mining concessions, and hydrographic networks (Instituto Geográfico Militar 2020; Agencia de Regulación y Control Minero 2025; Ministerio de Ambiente y Energía 2025). Spatial analyses were conducted to delimit the AOO and identify potential threats within the known distribution range.

## Taxonomic treatment

### Pleurothallis pembertonii

Taxon classificationPlantaeAsparagalesOrchidaceae

M.F.Monteros & Mark Wilson sp. nov.

5EB123AB-4D3A-539F-BBE3-ECFAF63CC509

urn:lsid:ipni.org:names:77382069-1

Figs 1, 2, 3, 5, 7a

#### Holotype.

Ecuador • Carchi, Reserva Dracula , 1965 m, 3 August 2021 (coordinates omitted for conservation reasons; detailed data on the herbarium type specimen). *Marco F. Monteros, MFM 217* (holotype: QCNE!).

**Figure 5.**
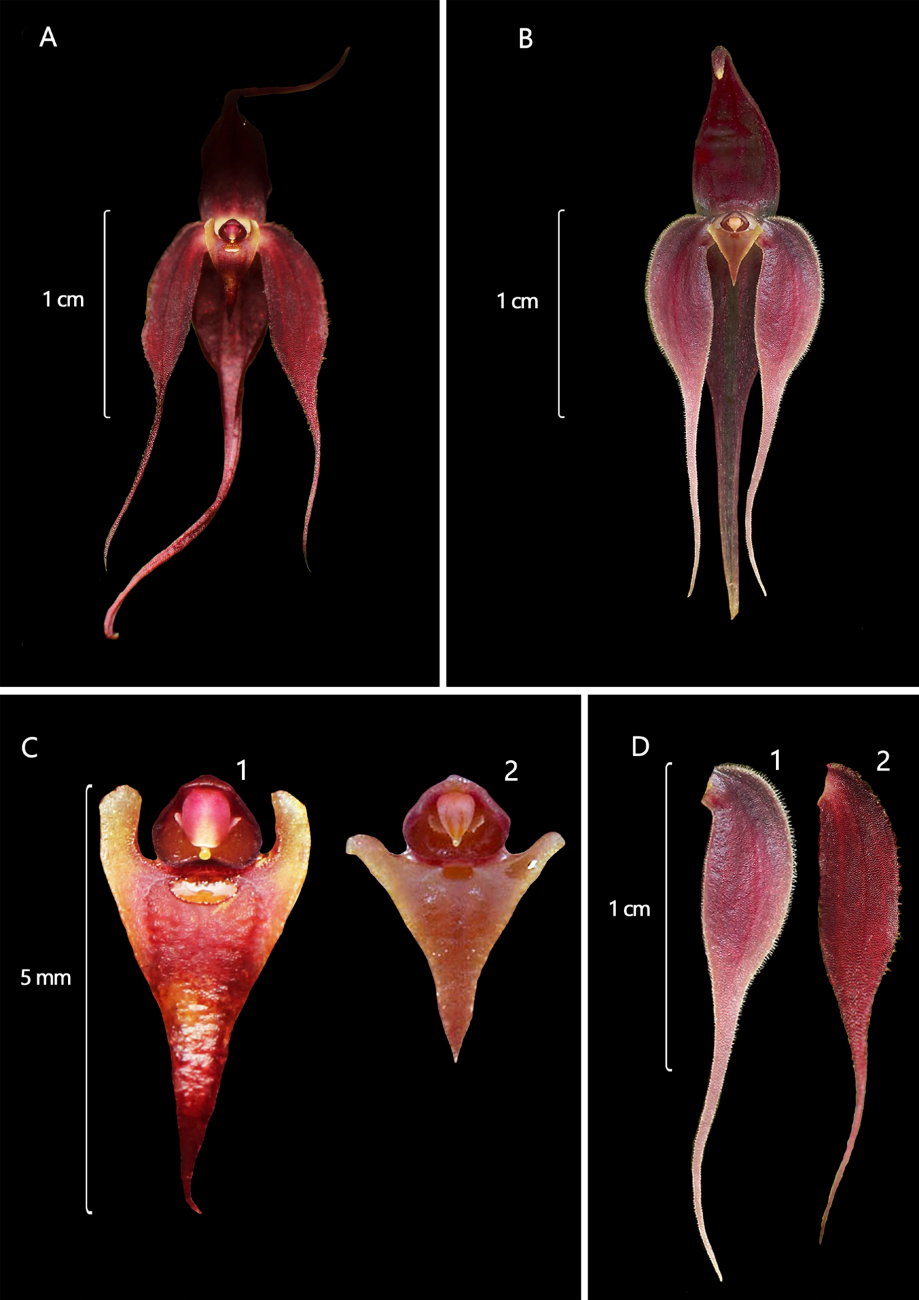
Comparison between *Pleurothallis pembertonii* M.F.Monteros & Mark Wilson and *Pleurothallis forceps-cancri* Luer & R.Escobar. **A**. *P. pembertonii*. **B**. *P. forceps-cancri*. **C1**. Lip, frontal view, *P. pembertonii*. **C2**. Lip, frontal view, *P. forceps-cancri*. **D1**. Petal of *P. pembertonii*. **D2**. Petal of *P. forceps-cancri*. Photographs by Marco F. Monteros (**A, C1, D1**) and Dale Borders (**B, C2, D2**).

**Figure 6.**
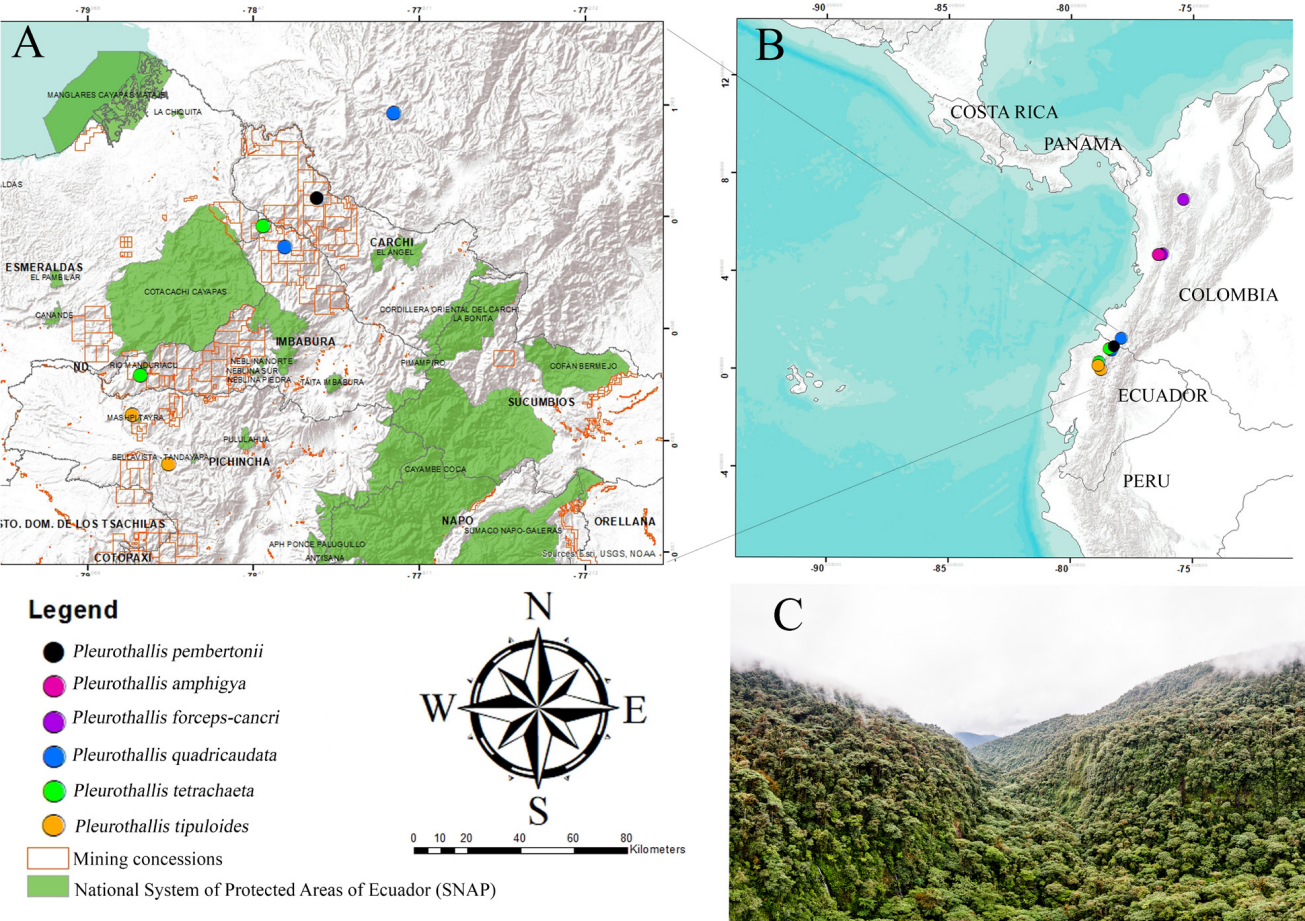
Distribution and conservation context of *Pleurothallis pembertonii* M.F.Monteros & Mark Wilson. **A**. Known distribution in northwestern Ecuador, showing the type locality and overlap with mining concessions. **B**. Geographic distribution of selected species of *Pleurothallis ser. Amphigyae* in Ecuador and Colombia. **C**. Habitat of *P. pembertonii* in lower montane cloud forest in Dracula Reserve. Photograph by Julio Carrión. Maps prepared by Marco F. Monteros.

**Figure 7.**
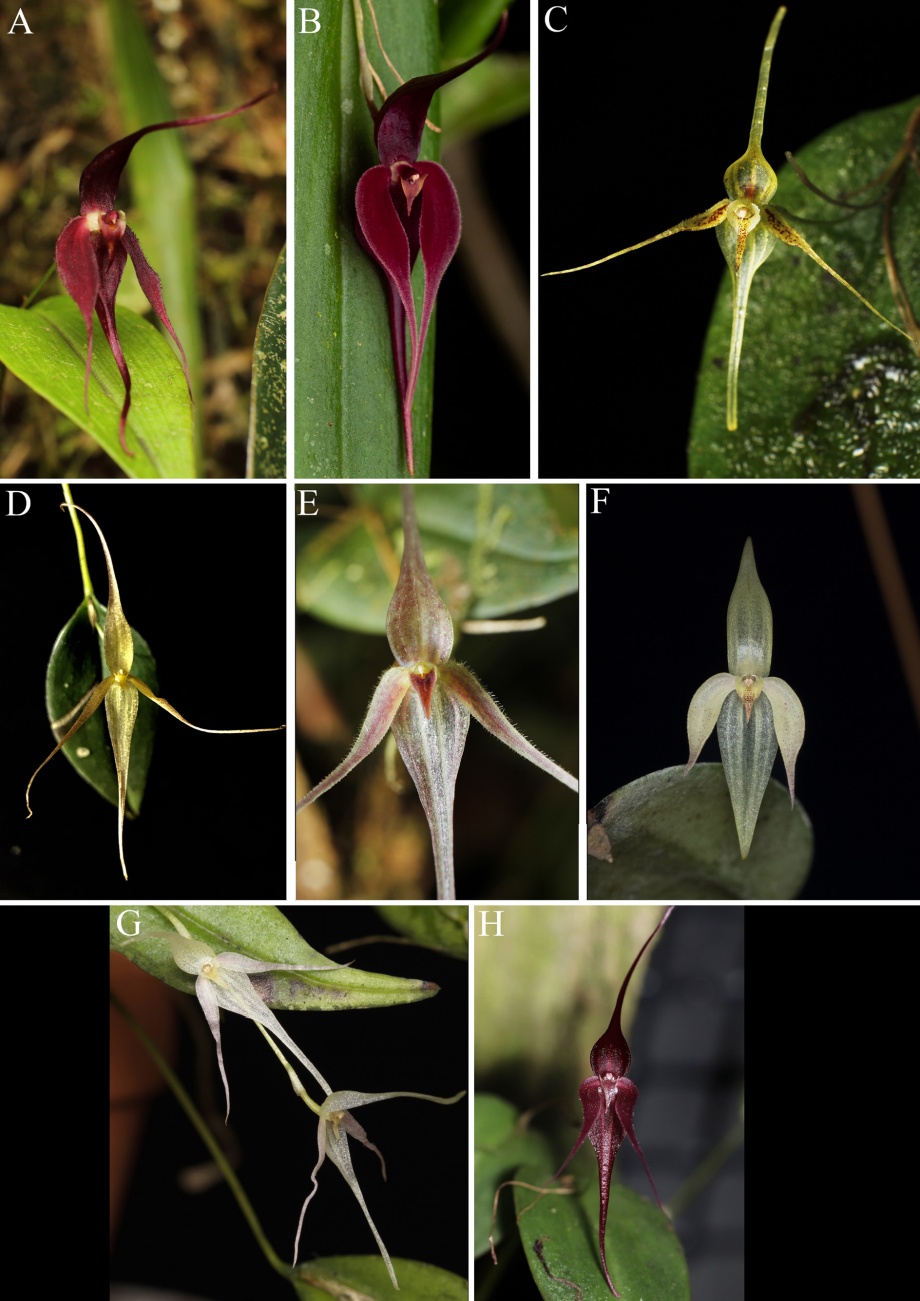
Morphologically similar species to *Pleurothallis pembertonii* M.F.Monteros & Mark Wilson. **A**. *P. pembertonii*; **B**. *P. forceps-cancri*; **C**. *P. tipuloides*; **D**. *P. quadricaudata*; **E**. *P. tetrachaeta*; **F**. *P. mundiflorae*; **G**. *P. lacrima*; **H**. *P. gymnastica*. Photographs by Marco F. Monteros (**A, C, E**), Sebastián Vieira (**B**), and Kevin Holcomb (**D, F, G, H**).

#### Diagnosis.

*Pleurothallis pembertonii* is most similar in appearance to *P. forceps-cancri* but differs in its oblong-subsigmoid petals (vs. elliptic-subsigmoid); sagittate-trilobed, attenuate lip, 5.0 × 2.5 mm (vs. triangular-trilobed lip, narrowly obtuse, 2.5–3.0 × 2.0–2.5 mm), and a reniform glenion at the base of the lip (vs. an orbicular glenion).

#### Description.

***Plant*** epiphytic, caespitose, medium-sized herb up to 12 cm tall; ***Roots*** slender ca. 0.5 mm in diameter. ***Ramicauls*** terete, suberect, slender, 7–8 cm long, 1 mm in diameter, provided with a 2 cm long tubular sheath at the base, papyraceous. ***Leaf*** suberect, coriaceous, rigid, sessile, elliptic-ovate, long-attenuate 5.0–6.0 × 1.5–2.0 cm. ***Inflorescence***, a single-flowered coflorescence, arising from the apex of the ramicaul and surrounded by a reclining, brown, papyraceous spathaceous bract when mature. ***Pedicel*** yellow-green, terete, 5 mm long. ***Ovary*** straight, canaliculate, 3 mm long. ***Flower*** sepals and petals vinaceous, the lip light purple with pale yellow lobes. ***Dorsal sepal*** glabrous, concave, ovate-lanceolate, long-attenuate, 16–17 × 3.0 mm, 3-veined. ***Lateral sepals*** connate into an ovate-lanceolate, long-attenuate synsepal, concave at the base, 19–20 × 5.0 mm, 4-veined. ***Petals*** descending in the natural position, oblong, oblique, subsigmoid, apex long-attenuate to acuminate, margins sparsely to densely ciliate, 16 × 3.0 mm, 3-veined. ***Lip*** sagittate-trilobed, 5.0 × 2.5 mm, the midlobe attenuate, the lateral lobes erect, rounded; glenion reniform, bearing two low, slightly verrucose, subparallel keels extending from the base to near the middle of the lip; base semi-reflexed; hinged to the base of the column. ***Column*** dark purple, semiterete, ventrally vesiculate, with prominent margins, stigma apical, bilobed, 1.2 × 1.5 mm. ***Anther cap*** broadly ovate, rose to pale yellow, 1.0 × 0.5 mm.

#### Additional material examined.

Ecuador • Carchi, Reserva Dracula , 1965 m, 10 July 2024. Marco F. Monteros, MFM 350 (paratype: QCNE!). Ecuador • Carchi, Reserva Dracula , 1970 m, 10 July. Marco F. Monteros, MFM 351 (QCNE). Ecuador. Carchi, Reserva Dracula , 1970 m, 10 July 2024. Marco F. Monteros, MFM 352 (QCNE).

#### Eponymy.

This species is named in honor of biologist Robert Pemberton, who contributed to the expansion of the Dracula Reserve and the protection of the orchid’s habitat.

#### Phenology and flower variations.

Plants of *Pleurothallis pembertonii* were observed flowering *in situ* in July and August. The flowers exhibit moderate variation in size, posture, and curvature of the floral segments (Fig. 3). The dorsal sepal ranges from nearly straight to distinctly arcuate or subsigmoid, and the degree of floral opening varies from widely spreading to more narrowly arranged. Flower coloration varies from deep vinaceous to lighter reddish tones, occasionally with yellowish hues toward the center of the flower. Nevertheless, the principal diagnostic characters remain stable throughout the population, including the oblong-subsigmoid petals, the sagittate-trilobed lip, the reniform glenion, and the pair of low, slightly verrucose, subparallel keels extending from the basal margins of the glenion toward the middle of the lip.

#### Distribution, habitat, and ecology.

*Pleurothallis pembertonii* grows epiphytically in lower montane cloud forests of northwestern Ecuador, at approximately 1970 m elevation, in a biologically important sector of the Tropical Andes Biodiversity Hotspot. According to the Ministerio del Ambiente del Ecuador (2013), the habitat where the species occurs corresponds to the Lower Montane Evergreen Forest of the Western Andes of Ecuador (BSBN04), an ecosystem characterized by high humidity, persistent cloud cover, a complex vertical forest structure, and exceptional levels of plant endemism. This ecosystem forms part of the western Andean foothills, a biogeographic transition zone between the Tropical Andes and the Chocó biogeographic region, where steep environmental gradients and historical isolation have promoted high rates of diversification and microendemism (Yánez-Muñoz et al. 2024).

The species is currently known only from the Dracula Reserve, a privately protected area managed by Fundación EcoMinga, which safeguards extensive tracts of well-preserved and highly threatened cloud forest. The occurrence of *P. pembertonii* within this reserve highlights the importance of private conservation initiatives in maintaining habitat continuity and protecting narrowly distributed epiphytic species.

*Pleurothallis pembertonii* occurs sympatrically with several other epiphytic orchid species characteristic of mature cloud forests, including *Dracula trigonopetala* Gary Mey. & Baquero, *Lepanthes quadricornis* Luer & R.Escobar, *L. tortilis* Luer & Hirtz, *L. longiacuminata* Luer & Hirtz, *L. tulcanensis* Baquero & Monteros, *Platystele finleyae* Monteros, E.Restrepo & Baquero, and *Pseudolepanthes bihuae* Monteros & Baquero (Luer and Escobar 1994; Luer 2005; Meyer et al. 2012). This assemblage reflects the high structural complexity and ecological stability of the forest and underscores the strong specialization of Pleurothallidinae to microhabitats associated with old-growth trees, sustained humidity, and minimal disturbance.

#### Preliminary conservation status.

The type locality and potential distribution area of *Pleurothallis pembertonii* are situated within the Mira–Mataje binational basin in northwestern Ecuador, a region of pronounced environmental heterogeneity and microendemism within the Tropical Andes Biodiversity Hotspot (Yánez-Muñoz et al. 2024). Despite more than a decade of intensive botanical exploration in northwestern Carchi and adjacent areas (Baquero and Iturralde 2017; Baquero and Zuchan 2017; Jiménez et al. 2018; Wilson et al. 2018; Baquero 2019; Baquero and Monteros 2020; Monteros et al. 2021, 2022), *P. pembertonii* is currently known from a single locality, indicating an extremely restricted distribution consistent with patterns observed in other narrowly endemic Pleurothallidinae.

The AOO, estimated at 4 km^2^, falls within the limits of the Critically Endangered (CR) category under subcriterion B2 (IUCN 2024). The principal threat to *P. pembertonii* is the extensive overlap of its habitat with active and proposed mining concessions, which promote forest loss, fragmentation, road opening, and long-term alteration of the humid microclimatic conditions essential for epiphytic orchids (Roy et al. 2018; INABIO 2025). Land-use change in montane forests has been shown to cause severe declines in vascular epiphyte richness and abundance, particularly affecting orchids that depend on mature host trees and stable humidity regimes (Krömer et al. 2025). Based on its extremely restricted AOO, occurrence at a single location, and the continuing decline in habitat quality due to ongoing anthropogenic pressures, *Pleurothallis pembertonii* is recommended for assessment as CR B2ab(iii) (IUCN 2024).

## Discussion

*Pleurothallis pembertonii* belongs to *Pleurothallis subg. Pleurothallis*, sect. *Pleurothallis*, subsect. *Acroniae*, ser. *Amphigyae*. It shares with other members of this series elongate, caudate sepals and petals; a single-flowered coflorescence; and a trilobed lip bearing a glenion (Luer 1986, 1998). In northwestern Ecuador and adjacent Colombia, species such as *P. amphigya*, *P. forceps-cancri*, *P. quadricaudata* Schltr., *P. tetrachaeta* Luer & Hirtz, and *P. tipuloides* Luer exhibit broadly similar floral architecture, reflecting a strong morphological affinity among species of the series. As illustrated by the comparative distribution map (Fig. 6), these taxa are largely restricted to the western Andean foothills and lower montane forests, mostly along the Ecuador–Colombia border region, an area recognized for high levels of endemism and rapid lineage diversification driven by complex topography and steep environmental gradients (Yánez-Muñoz et al. 2024). This shared morphology, particularly the extreme attenuation of the sepals and petals, has historically complicated species delimitation within the group and has resulted in repeated misidentifications.

Despite these shared traits, *Pleurothallis pembertonii* is consistently distinguished by a unique combination of vegetative and floral characters summarized in Table 1. Unlike species with extremely narrow, filiform floral segments, such as *P. quadricaudata*, *P. tetrachaeta*, and *P. tipuloides*, *P. pembertonii* exhibits ovate-lanceolate dorsal and lateral sepals that are long-attenuate but proportionally broader (Fig. 7). In comparison with *P. amphigya* and *P. forceps-cancri*, *P. pembertonii* is readily separable by its oblong, oblique, subsigmoid petals that are long-attenuate and by a sagittate-trilobed lip bearing two small divergent keels and a well-developed reniform glenion (Fig. 5). These characters remain stable across all examined individuals and do not overlap with those observed in related taxa. Additional related species, such as *P. gymnastica* K.W.Holcomb, *P. lacrima* K.W.Holcomb, *P. mark-wilsonii* J.S.Moreno, Gal-Tar & Sierra-Ariza and *P. mundiflorae* K.W.Holcomb, can also be distinguished from *P. pembertonii* by differences in the morphology of the lip and the degree of development of the glenion (Fig. 7). In addition, the latter species is readily separated by the presence of a markedly involute dorsal sepal and strongly revolute petals (Moreno et al. 2022).

**Table 1.** Diagnostic morphological differences between *Pleurothallis pembertonii* M.F.Monteros & Mark Wilson and allied species of *Pleurothallis ser. Amphigyae*.

	***Pleurothallis pembertonii***	***P. amphigya***	***P. forceps-cancri***	***P. gymnastica***	***P. lacrima***	***P. mark-wilsonii***	***P. quadricaudata***	***P. tetrachaeta***	***P. tipuloides***
**Dorsal sepal**	ovate-lanceolate, long-attenuate (17 × 3.0 mm)	ovate, acute, long-attenuate (20–30 × 4.0 mm)	ovate, acute, acuminate (20 × 6.0 mm)	Long-attenuate, acute (21 × 5.0 mm)	very narrowly ovate, long-attenuate, acute (11 × 4.0 mm)	ovate-lanceolate, very involute forming a tube, acute, long-attenuate (30–35 × 8.0–13 mm)	very narrowly ovate, acute, long-attenuate (23–50 × 3.0–6.0 mm)	very narrowly ovate, acute, long-attenuate (25–40 × 2.3–3.0 mm)	narrowly ovate, acute long-attenuate (28 × 3.0 mm)
**Lateral sepal**	ovate-lanceolate, long-attenuate (19–20 × 5.0 mm)	ovate, acute, long-attenuate (20–32 × 3.5–5.0 mm)	ovate, acute, acuminate (16 × 4.0 mm)	Long-attenuate, acute (21 × 5.0 mm)	very narrowly ovate, long-attenuate, acute (11 × 4.0 mm)	ovate-lanceolate, very involute forming a tail, acute, long-attenuate (38–40 × 6.0–9.0 mm)	very narrowly ovate, acute, long-attenuate (23–50 × 3.0 mm)	very narrowly ovate, acute, long-attenuate (25–40 × 3.5–4.0 mm)	narrowly ovate, acute long-attenuate (28 × 3.0 mm)
**Petals**	oblong, oblique, subsigmoid, long-attenuate-acuminate (16 × 3.0 mm)	elliptical-ovate or subsigmoid, oblique, acute, slightly acuminate (8–10 × 2.0–2.5 mm)	elliptical-subsigmoid, oblique, acute, acuminate (18 × 4.0 mm)	elliptical, subsigmoid, oblique, acute (20 × 4.0 mm)	very narrowly ovate, long-attenuate, acute (9.0 × 2.5 mm)	oblong-lanceolate, revolute, strongly acuminate (17–20 × 2.0–4.0 mm)	very narrowly ovate, acute, subsigmoid, long-attenuate (23–45 × 3.0 mm)	very narrowly ovate, acute, subsigmoid, long-attenuate (20–35 × 1.5–1.75 mm)	narrowly ovate, subsigmoid, acute, long-attenuate (20 × 1.5 mm)
**Lip**	sagittate-trilobed, attenuate-acuminate, (5.0 × 2.5 mm), the disc with two diverging small keels and a reniform glenion	triangular-trilobed, acute (2–3 × 1.5–2.5 mm), with a glenion	triangular-trilobed, narrowly obtuse (2.5–3.0 × 2.0–2.5 mm), the disc with an orbicular glenion.	narrowly triangular – trilobed, acute (5.0 × 1.5 mm), with a very shallow, poorly defined glenion	triangular-trilobed, acute (3.0 × 1.5 mm), with a small glenion	triangular, trilobed, curved at the basal part (view laterally), acuminate (3.2–3.5 × 2.5–2.8 mm), with two diverging keels, elongated	triangular-sagittate, (3.5–4.0 × 1.5–2.5 mm), with a glenion.	triangular-sagittate (2.5 × 1.0 mm)	ovate-trilobed (5.0 × 2.0 mm), with a small glenion.

Finally, the case of *Pleurothallis pembertonii* highlights a recurrent problem in Pleurothallidinae taxonomy: the persistent confusion of morphologically similar species circulating in cultivation and trade under misapplied names. As documented for *Pleurothallis*, *Dracula*, *Trisetella*, and *Porroglossum*, many species have entered international markets before formal taxonomic resolution, often lacking reliable locality data (Baquero and Mogrovejo 2021; Parra-Sánchez et al. 2024; Baquero et al. 2025). In cloud forest ecosystems already under intense pressure from mining concessions, deforestation, and land-use change, such taxonomic inaccuracies have direct conservation consequences, as microendemic epiphytic species may remain unrecognized, unassessed, or mismanaged if treated as widespread taxa (Roy et al. 2018; Krömer et al. 2025). Rigorous taxonomic treatment is essential for effective conservation because misidentifications can obscure microendemic epiphytic species in highly threatened cloud forests, compromising their proper assessment and management.

## Supplementary Material

XML Treatment for Pleurothallis pembertonii

## Additional information

### Artificial Intelligence (AI) use

The authors accept full responsibility for the content of the manuscript, including the disclosure of any use of AI.

No AI tools were used in the preparation of this manuscript.

### Author ORCIDs

M.F. Monteros https://orcid.org/0000-0002-6670-3687

K. Holcomb https://orcid.org/0000-0002-0571-4462

L.E. Baquero https://orcid.org/0000-0002-1444-5727

M. Wilson https://orcid.org/0000-0003-3044-7471

### Data availability

All of the data that support the findings of this study are available in the main text.
